# MicroRNA-495 confers inhibitory effects on cancer stem cells in oral squamous cell carcinoma through the HOXC6-mediated TGF-β signaling pathway

**DOI:** 10.1186/s13287-020-1576-3

**Published:** 2020-03-14

**Authors:** Xiaolong You, Zhengyu Zhou, Wen Chen, Xiaoyong Wei, Heqiang Zhou, Wenzheng Luo

**Affiliations:** 1 Department of Head and Neck Surgery, Jiangxi Cancer Hospital, No. 519, Beijing East Road, Nanchang, 330029 Jiangxi Province People’s Republic of China; 2 Department of Intensive Care Medicine, Jiangxi Cancer Hospital, Nanchang, 330029 People’s Republic of China; 3 Department of Plastic Surgery, Jiangxi Cancer Hospital, Nanchang, 330029 People’s Republic of China; 4 Department of Liver Oncology, Jiangxi Cancer Hospital, Nanchang, 330029 People’s Republic of China

**Keywords:** microRNA-495, Oral squamous cell carcinoma, Cancer stem cells, Homeobox C6, TGF-β signaling pathway, Migration, Invasion, Epithelial-mesenchymal transition

## Abstract

**Background:**

Oral squamous cell carcinoma (OSCC) is associated with high morbidity and ranks sixth among malignancies worldwide. Increasing evidence suggests that microRNAs (miRNAs or miRs) play a critical role in regulating cancer stem cells (CSCs), which drive the proliferation and spread of OSCC. Therefore, based on the alteration of aberrantly expressed miR-495 and homeobox C6 (HOXC6) by Gene Expression Omnibus (GEO) analysis, we subsequently explore the potential effect of miR-495 on the progression of CSCs in OSCC.

**Methods:**

After the isolation of CSCs from the clinical tissue samples of OSCC patients, the expression of miR-495 and HOXC6 was determined, followed by the validation of the relationship between miR-495 and HOXC6. Subsequently, gain- and loss-function approach was performed to detect the role of miR-495 and HOXC6 in cell proliferation, migration, invasion, cell cycle entry, apoptosis, and epithelial-mesenchymal transition (EMT) of CSCs in OSCC, as well as the tumor growth in vivo.

**Results:**

HOXC6 was highly expressed while miR-495 was poorly expressed in OSCC. HOXC6 was verified to be a target gene of miR-495, and miR-495 could inhibit the activation of the TGF-β signaling pathway. CSCs with miR-495 overexpression or HOXC6 silencing exhibited reversed EMT process; reduced abilities of proliferation, migration, and invasion; and promoted cell apoptosis in vitro. Moreover, inhibited tumor growth was observed in vivo after injection with miR-495 agomir or sh-HOXC6. In contrast, the downregulation of miR-495 showed an induced role in the progression of OSCC.

**Conclusion:**

These findings suggest that miR-495 may suppress HOXC6 to inhibit EMT, proliferation, migration, and invasion while promoting apoptosis of CSCs in OSCC by inhibiting the TGF-β signaling pathway.

## Background

Oral squamous cell carcinoma (OSCC) is one of the most prevalent types of malignant tumor in the oral system. The 5-year survival rate of patients with OSCC is as low as 60% and has scarcely improved over the last 15 years [1]. The likelihood of recurrent OSCC is associated with several predictive factors including tumor stage, depth of tumor penetration, positive surgical margins, extracapsular spread, and perineural invasion [2]. A small subpopulation of cancer stem cells (CSCs) have been identified in OSCC based on their ability for perpetual self-renewal and proliferation, producing downstream progenitor cells and cancer cells that drive tumor growth [3, 4]. Therefore, increasing attention is being given to the role of CSCs in the progression of OSCC and their potential as targets for cancer treatment [5].

MicroRNAs (miRNAs or miRs), endogenous, and small non-coding single-stranded RNAs of 22 nucleotides that involved in diverse biological processes, including cell cycle, growth, and apoptosis, regulate gene expression by targeting multiple molecules at the posttranscriptional level [6]. Over the past 10 years, many in vivo and in vitro experiments have confirmed the importance of miRNAs in cancer initiation, progression, and metastasis [7]. Moreover, the dysregulation of miRNAs contributes to CSC tumorigenicity, asymmetric cell division, and chemoresistance [8]. Increasing evidence suggests that aberrant expression of miR-495 is associated with the progression of various cancers. A recent study suggests that miR-495 may serve as a tumor suppressor by inhibiting the migration and invasion of gastric cancer cells [9]. In addition, restored miR-495 expression can restrict cell proliferation and invasion in OSCC cells in vitro by repressing its target gene Notch1 [10]. The HOX gene family consists of 39 genes that organize into 4 chromosomal loci, which has been reported to play a significant role in organogenesis via regulation of proliferation, differentiation, survival, migration, and invasion [11]. Homeobox C6 (HOXC6) can be used as a prognostic marker in patients with esophageal squamous cell carcinoma since the median survival time of patients with high HOXC6 expression is found to be poor [12]. Moreover, OSCC patients with lymph node metastasis present with high expression of HOXC6 [13], suggesting the implication of aberrant HOX gene expression in the development of OSCC. Silencing of HOXC6 is capable of inhibiting epithelial-mesenchymal transition (EMT) through the inactivation of the transforming growth factor-beta (TGF-β)/Smad signaling pathway in cervical carcinoma cells [14]. TGF-β encompasses TGF-β1, 2, and 3 and has been reported to play a significant role in the regulation of cancer by suppressing tumor growth in the early stage but promoting tumor growth in established cancers [15]. High TGF-β expression at tumor center predicts poorer overall survival and recurrence-free and disease-free survival in OSCC patients [16]. However, the reports on the interaction among miR-495, HOXC6, and the TGF-β signaling pathway in the development of OSCC remain enigmatic. Therefore, the present study aimed to investigate the role of miR-495 as a novel tumor suppressor and its effect on the cell proliferation, migration, and invasion of CSCs in OSCC, which may provide a novel therapeutic target for the treatment of OSCC in the future.

## Materials and methods

### Ethics statement

The study was conducted under the approval of the Institutional Review Board of Jiangxi Cancer Hospital. Informed written consent was obtained from each participant prior to the study. Animal experiments were conducted under the approval of the Animal Ethics Committee of Jiangxi Cancer Hospital.

### Microarray-based gene expression profiling

The OSCC-related gene expression datasets were retrieved from the Gene Expression Omnibus (GEO) database (https://www.ncbi.nlm.nih.gov/geo/). Then, the GSE31853, GSE74530, and GSE30784 datasets were obtained (all contained different normal control samples and OSCC samples) and used for differential analysis. The samples in the three datasets were subjected to differential analysis by the “limma” package and “input” package of R language software, with the screening threshold of |logFC| > 2 and *p* value < 0.05. Dataset GSE37991 was also obtained, which contained 40 normal control samples and 40 OSCC samples. The expression of the screened differentially expressed genes (DEGs) was searched from the dataset, and their expression was drawn as a boxplot using the R language.

TargetScan database (http://www.targetscan.org/vert_71/), DIANA database (http://diana.imis.athena-innovation.gr/DianaTools/index.php?r=microT_CDS/index), mirDIP database (http://ophid.utoronto.ca/mirDIP/index.jsp#r), and microRNA.org database (http://34.236.212.39/microrna/home.do) were employed for gene screening. In the four datasets, prediction on the miRNAs regulating HOXC6 was performed by setting “HOXC6” as the input and “human” as the species. The predicted results were screened according to the scores, followed by intersection analysis to identify the study subjects for the follow-up study.

A Venn diagram was constructed using the website (http://bioinformatics.psb.ugent.be/webtools/Venn/), which was used to identify the intersection of the screened results from different datasets. Elements of the different datasets were included in the website, and the names of these datasets were provided. The Venn diagram was constructed using the website, and the intersection of the different datasets was identified.

### Purification and identification of OSCC cells

Sixty cases of fresh OSCC tissue samples were collected after the surgical resection in the maxillofacial surgery of Jiangxi Cancer Hospital (Nanchang, Jiangxi, China) and used for sample selection and pre-treatment. Anti-pollution treatment for samples was then conducted. In brief, deep tissues of about 1.0 × 1.0 × 0.5 cm^3^ in size were washed with 0.9% saline containing 250 mL of 1.6 million units penicillin until the tissues turned white for 3 to 4 times. Then, the tissues were immersed in saline vials, sealed tightly, and sent to the laboratory.

For culture and purification of OSCC cells, the tissues were placed in Petri dishes and cut to expose the fresh tissues. Then, the fresh tissues were rinsed twice with sterile phosphate buffer saline (PBS), added with serum-free culture medium, and cut into the size of about 1 mm^3^ and tissue debris. A total of 3 mL of tissue and culture medium was transferred into small test tubes and added with collagenase IV, followed by detachment by culture in a CO_2_ incubator for about 1 h. Then, the detached tissues were centrifuged at 1500 rpm using a low-speed centrifuge with the supernatant discarded. Next, 3 mL of culture medium was added to the tissues, and they were repeatedly triturated with a pipette and centrifuged at a low speed, with the supernatant discarded. The procedure was repeated twice. After the addition of 4 mL of Iscove’s modified Dulbecco’s medium (IMDM) containing 10% serum, the cells were seeded into the first two wells of 6-well plates and added with IMDM until the volume reached 3 mL, followed by culture in a CO_2_ incubator. After 24 h, the adherent cells were detached using 0.25% trypsin and inoculated. The non-adherent tissues and cells were transferred to the next well every 30 min. The cells from the first two wells that were filled with 3 mL of IMDM containing 10% serum were collected and cultured in an incubator after repeated adherence achieved on the 6th wells.

### Observation and the change of medium

After 12 h of incubation, most of the cells and tissues were firmly adhered to wall and cells expanded and became larger. After 24 h of culture, the medium was replaced and the non-adherent cells and tissues were gently removed, while the remaining cells were cultured in the new medium.

### Repeated adherence and differential culture for a second time

When cells reached about 80% confluence, they were treated with trypsin and removed from the third row of wells to a new 6-well plate. After repeated adherence and culture, the relatively purified OSCC cells were obtained.

The identification of OSCC cells was performed. In short, immunohistochemistry was applied to stain keratin, Vimentin, CSC-related identification, and isolation markers BMI1, ALDH1, and CD44. Slides containing OSCC cells were prepared on the strictly sterilized coverslips. The staining was performed following the manufacturer’s instructions of the streptavidin peroxidase (SP) immunohistochemistry kit, with polyclonal antibodies purchased from Abcam (Cambridge, UK) used as the primary antibodies: keratin (1:1000, ab155478), Vimentin (1:1000, ab16700), BMI1 (1:1000, ab155478), ALDH1 (1:1000, ab155478), and CD44 (1:1000, ab155478). Positive and negative controls were prepared during staining. Known positive cell slides were used as the positive control, while PBS was used to replace the primary antibody as the negative control (NC). The cytoplasm and/or cell membrane showing tan-stained particles was considered positive.

### Sorting of CSCs in OSCC

The CSCs at logarithmic growth phase were collected and adjusted to a density of 1 × 10^7^ cells/mL. Cells were then incubated with anti-CD133-PE (566,593, 1:500, Becton, Dickinson and Company, NJ, USA) (with absorbed light of 490–560 nm and emission wavelength of 595 nm) and anti-CD44-fluorescein isothiocyanate (FITC) (555,478, 1:500, Becton, Dickinson and Company, NJ, USA) (with excitation wavelength of 495 nm and emission wavelength of 519 nm) for 30 min at room temperature. The cells were subsequently resuspended in 1 mL of PBS, filtered through a 40-μm aseptic screen mesh and placed on ice for analyzing and sorting. The isotype control antibody was used to label cells under the same conditions. Cells were then sorted using a FACSAria II flow cytometer (Becton, Dickinson and Company, NJ, USA) for detection, followed by fluorescence labeling using different antibodies, while the CD133 and CD44 double-positive cells to be sorted were circled. Lastly, the circled cells were sorted aseptically into sorting tubes.

### CSC suspension sphere-forming test

The sorted CD133 and CD44 double-positive cells were seeded into low-adsorptive 96-well plates and cultured with serum-free Dulbecco’s modified Eagle’s medium (DMEM)-F12 containing 20 ng/mL epithelial growth factor (EGF) and 20 ng/mL fibroblast growth factor (FGF)-β for 10 consecutive days, with the medium changed semi-quantitatively every 2 days. Lastly, the cells were observed, photographed, and counted under a CKX41 inverted optical microscope (Olympus Optical Co., Ltd., Tokyo, Japan).

### Dual-luciferase reporter assay

The Bioinformatics Database (microRNA.Org; http: //www.microrna.org/) was employed to predict whether HOXC6 was a target gene of miR-495. Human embryonic kidney HEK293T cells were cultured in DMEM containing 10% fetal bovine serum (FBS) with 5% CO_2_ at 37 °C. The complementary DNA (cDNA) fragment of HOXC6 3′-untranslated region (3′-UTR) containing the miR-495 binding site was inserted into the pmirGLO vector. The cDNA fragment of HOXC6 3′-UTR with the mutated binding site was constructed by site-directed mutagenesis and inserted into the pmirGLO vector. The pmirGLO-HOXC6 (WT) or pmirGLO-mutant type (Mut) HOXC6 recombinant vector was then co-transfected into HEK293T cells with miR-495 mimic (miR-495 overexpression sequence) or miR-NC (NC sequence) by lipofection. After 48 h of culture, the cells were collected and lysed. An amount of 100 μL of Renilla luciferase detection working solution was added to 100 μL of lysate supernatant, followed by the detection of Renilla luciferase activity. In addition, 100 μL of firefly luciferase detection reagent was mixed with 100 μL of lysate supernatant before the detection of firefly luciferase activity. A multi-functional microplate reader SpectraMax M5 was used to assess the activity of Renilla luciferase and firefly luciferase, respectively, with interval time set as 2 s and the detection time as 10 s.

### Cell treatment

The CSCs were cultured in high-glucose DMEM containing 10% FBS in a 5% CO_2_ incubator at 37 °C with saturated humidity. The cells were sub-cultured when cells reached approximately 75% confluence. The cells in logarithmic growth phase were treated with trypsin and added with 1.4 mL of PBS to adjust cell density to 2 × 10^5^ cells/mL. Cells were then assigned into 6 groups: blank (CSCs without transfection), NC (CSCs transfected with scramble small interfering RNA [siRNA]), miR-495 mimic (CSCs transfected with miR-495 mimic, purchased from Invitrogen Inc., Carlsbad, CA, USA), miR-495 inhibitor (CSCs transfected with miR-495 inhibitor, purchased from Sigma-Aldrich Chemical Company, St Louis, MO, USA), si-HOXC6 (CSCs transfected with silenced HOXC6, purchased from Shanghai GenePharma Co., Ltd., Shanghai, China), and miR-495 inhibitor + si-HOXC6 (CSCs transfected with miR-495 inhibitor and added with 100 ng/mL silenced HOXC6 to stimulate cells). The sequences of transfected primers are listed in Table 1. After 6 h of transfection, the medium was changed, after which cells were then incubated for 48 h, and collected for subsequent experiments. Human OSCC cells in logarithmic growth phase were seeded into 6-well plates. When cells reached about 30–50% confluence, cells were transfected according to the manufacturer’s instructions of lipofectamine 2000 (Invitrogen, Carlsbad, CA, USA). About 100 pmol of miR-495 mimic, miR-495 inhibitor, si-HOXC6, and their NCs (final concentration was 50 nM) were diluted in 250 μL of serum-free Opti-minimum essential medium (MEM) (Gibco, Grand Island, NY, USA), mixed well, and incubated at room temperature for 5 min. Next, 5 μL of lipofectamine 2000 was mixed with 250 μL of serum-free medium Opti-MEM and incubated for 5 min at room temperature. The above two solutions were then mixed together and incubated at room temperature for 20 min, followed by addition to the cell culture wells. After incubation at 37 °C with 5% CO_2_ for 6–8 h, cells were then incubated with DMEM containing 10% FBS and penicillin/streptomycin for 24–48 h before subsequent experiments.

**Table 1:** Sequences of transfection primers

Groups	Primer sequences (5′-3′)
siRNA-NC	UUCUUCUAACUUUUCACGUUC
miR-495 mimic	AAAGAACACTTTTCGGTATT
miR-495 inhibitor	AATACCGAAAAGTGTTCTTT
siRNA-HOXC6	UUUAAUAUUUAUUUCUGUCUC

*siRNA* small interfering RNA, *HOXC6* homeobox C6, *NC* negative control, *miR-495* microRNA-495

### Reverse transcription-quantitative polymerase chain reaction

Total RNA was extracted from cells of each group using RNA extraction kit (Invitrogen, Carlsbad, CA, USA), followed by determination of RNA concentration. Primers for miR-495, HOXC6, TGF-β, TGFβRI, TGFβRII, Smad4, Vimentin, E-cadherin, N-cadherin, glyceraldehyde phosphate dehydrogenase (GAPDH), and U6 were designed and synthesized by Takara (Takara Holdings Inc., Kyoto, Japan) (Table 2). Then, the total RNA was reverse transcribed into cDNA according to the manufacturer’s instructions of reverse transcription kit (DRR047S, TaKaRa Co., Ltd., Dalian, China). RT-qPCR was conducted using ABI 7500 instrument (ABI Company, Oyster Bay, NY). U6 was used as an internal reference for the relative expression of miR-495, while GAPDH was used as an internal reference for the relative expression of HOXC6, TGF-β, TGFβRI, TGFβRII, Smad4, Vimentin, E-cadherin, and N-cadherin. The fold changes were calculated using the relative quantification (2^−ΔΔCt^ method) [17].

**Table 2:** The primer sequences for RT-qPCR

Gene	Sequence (5′-3′)
miR-495	Forward: GCGCGTGAGCAGGCTGGAGAAATT Reverse: AAACAAACATGGTGCA
HOXC6	Forward: ACAGACCTCAATCGCTCAGGA Reverse: AGGGGTAAATCTGGATACTGGC
TGF-β	Forward: CAACGCCATCTATGAGAAAACC Reverse: AAGCCCTGTATTCCGTCTCC
TGFβRI	Forward: ATTACCTGGACATCGGCA AC Reverse: TTGGGCACCACATCATAGAA
Smad4	Forward: GTGGCTGGTCGGAAAGGATT Reverse: ACTGGCAGGCTGACTTGTGG
TGFβRII	Forward: ACTTGACCTGTTGCCTGTGTGAC Reverse: CTGGCTTCAACGCCTTTCACCTCA
N-cadherin	Forward: TCATTGCCATCCTGCTCTGCAT Reverse: AGTTGTTTGGCCTGGCGTTCTT
Vimentin	Forward: AAAGTGTGGCTGCCAAGAACCT Reverse: ATTTCACGCATCTGGCGTTCCA
E-cadherin	Forward: TTAGGTTAGAGGGTTATCGCGT Reverse: TAACTAAAAATTCACCTACCGACC
U6	Forward: CGCTTCACGAATTTGCGTGTCAT Reverse: GCTTCGGCAGCACATATACTAAAAT
GAPDH	Forward: ACAGTCAGCCGCATCTTCTT Reverse: GACAAGCTTCCCGTTCTCAG

*RT-qPCR* reverse transcription quantitative polymerase chain reaction, *miR-495* microRNA-495, *HOXC6* homeobox C6, *TGF-β* transforming growth factor β, *TGF-βRI* transforming growth factor βreceptor I, *TGF-βRII* transforming growth factor βreceptor II, *Smad4* sma and mother against ddp four, *GAPDH* glyceraldehyde-3-phosphate dehydrogenase, *U6* U6 snRNA

### Western blot analysis

Total protein was extracted from cells and tissues using radio-immunoprecipitation assay (RIPA) lysis buffer (Gibco, Carlsbad, CA, USA) containing phenylmethanesulfonyl fluoride (PMSF), and protein concentration was determined using the Bradford method. After separation with 10% sodium dodecyl sulfate-polyacrylamide gel electrophoresis (SDS-PAGE), the proteins were subsequently transferred onto polyvinylidene fluoride (PVDF) membranes. The membranes were blocked with Tris-buffered saline with Tween 20 (TBST) containing 5% skim milk powder for 1 h, and then incubated overnight at 4 °C with the following primary antibodies: CD133 (1:500, ab19898, Abcam), CD44 (1:2000, ab157107, Abcam), HOXC6 (1:1000, ab191542, Abcam), TGF-β (1:1000, ab190503, Abcam), TGFβRI (1:1000, ab31013, Abcam), TGFβRII (1:1000, ab78419, Abcam), Smad2 (1:1000, 5339, Cell Signaling Technology), Smad4 (1:5000, ab40759, Abcam), Smad7 (1:1000, ab90086, Abcam), N-cadherin (1:1000, ab18203, Abcam), Vimentin (1:1000, ab92547, Abcam), Ecadherin (1:50, ab1416, Abcam), and GAPDH (internal reference, 1:5000, Santa Cruz Biotechnology Inc., CA, USA). Subsequently, the membrane was incubated with horseradish peroxidase-labeled secondary antibody at room temperature for 2 h. Finally, the membrane was developed using autoradiography, photographed, and recorded, after which the film was scanned. Quantification analysis was performed using Gel-Pro Analyzer 4.0, and the ratio of the gray value of target bands to that of internal reference bands served as the relative expression of proteins.

### 3-(4,5-Dimethylthiazol-2-yl)-2,5-diphenyltetrazolium bromide (MTT) assay

The isolated and purified CSCs were seeded into 96-well plates and grouped into the blank, NC, miR-495 mimic, miR-495 inhibitor, si-HOXC6, and miR-495 inhibitor + si-HOXC6 groups. Cells in each well were added with 20 μL of MTT (5 mg/mL) at 0, 24, 48, and 72 h, respectively, followed by 4 h of incubation at 37 °C after which the supernatant was discarded. Then, 200 μL of dimethyl sulfoxide (DMSO) was added to the cells to dissolve the bluish-violet precipitate. The optical density (OD) value was then measured at 570 nm.

### Scratch test

The effect of miR-495 expression on CSC migration was evaluated by scratch test. After 24 h of transfection, the cells were seeded into 6-well plates. Once cells reached full confluence, a 10-μL pipette tip was used to gently scratch along the central axis of the plate and the floating cells were washed away with PBS. After 24 h of culture, the cells were observed and photographed under a microscope and the cell migration was expressed as the healing percentage of scratched cells. The percentage of cell healing = (scratch width before experiment − scratch width after culture for 24 h)/scratch width before experiment × 100%.

### Transwell assay

Basement membranes of the Transwell chambers were coated using Matrigel. The cells in each group were collected after 24 h of transfection and resuspended in serum-free medium for 16 h to allow cell starvation. The apical chamber was added with cell suspension at a density of 1 × 10^5^, followed by the addition of culture medium containing 10% FBS to the basolateral chamber. After 24 h of incubation in a 5% CO_2_ incubator at 37 °C, cells failing to invade were discarded, while the remaining cells were fixed with 4% paraformaldehyde and stained with 0.5% crystal violet. At last, the number of cells passing through the membrane was calculated in five randomly selected visual fields under a microscope, with the average value obtained.

### Flow cytometry

Flow cytometry was used to detect the effect of miR-495 on the apoptosis of CSCs. Cell apoptosis was detected by Annexin V-FITC/propidium iodide (PI) double-staining cell apoptosis detection kit (40303ES20, Yeasen Biotechnology Co. Ltd., Shanghai, China). Annexin V+ and PI− cells were considered as apoptotic cells. The percentage of early apoptotic cells (%) = Annexin V-FITC positive cells/total cells × 100%.

The cells in the blank group and the miR-495 mimic group after 24 h of treatment were centrifuged, collected, washed three times with pre-cooled PBS, and centrifuged at 1000 rpm for 5 min at room temperature with the supernatant discarded. Cells in the tube were added with 1 mL of pre-cooled 70% ethanol and fixed overnight at 4 °C. The next day, cells were centrifuged at 700 rpm for 5 min at room temperature, resuspended in 0.5 mL of PBS, added with RNase A (final concentration of 50 μg/mL), and allowed to stand for 60 min at room temperature. Cells were then stained with 500 μL of PI (100 μg/mL) and incubated for 30 min in the dark. CellQuest was finally used to detect cells at the G0/G1, S, and G2/M phases.

### Xenograft tumor in nude mice

A total of 36 female-specific pathogen-free (SPF) grade BALB/C nude mice (4–6 weeks old) (Animal Center of Sichuan University, Chengdu, Sichuan, China) were used in this study. The feeding of nude mice and all animal experiments were performed in the animal center. The successfully constructed lentiviral vectors were subcutaneously injected into nude mice using a 1-mL syringe. The nude mice were assigned into the blank (without lentiviral vector injection), NC (injected with NC lentiviral vector), miR-495 agomir (injected with miR-495 agomir lentiviral vector), miR-495 antagomir (injected with miR-495 antagomir lentiviral vector), short hairpin RNA (sh)-HOXC6 (injected with sh-HOXC6 lentiviral vector), and miR-495 inhibitor + sh-HOXC6 (injected with miR-495 inhibitor and sh-HOXC6 lentiviral vector) groups. After inoculation of the lentiviral vector, all nude mice were fed in the laminar hood of SPF animal room. The long diameter (*L*) and short diameter (*W*) of the tumor were measured every 7 days. The tumor volume (*V*) = 1/2 × *LW*2 was calculated, and the tumor growth curve was drawn. On the 28th day, the nude mice were euthanized using cervical dislocation method and their xenografts were removed for photographing and comparison.

### Statistical analysis

Statistical analyses were conducted using the SPSS 21.0 statistical software (IBM Corp. Armonk, NY, USA). Measurement data in accordance with normal distribution were expressed as mean ± standard deviation. Data between two groups were compared by independent sample *t* test, and data among multiple groups were compared by one-way analysis of variance (ANOVA). Pairwise comparisons were conducted by least significant difference (LSD) test. A value of *p* < 0.05 was considered statistically significant.

## Results

### Bioinformatics analysis predicting the DEGs and their molecular interactions in OSCC

**Fig. 1:**
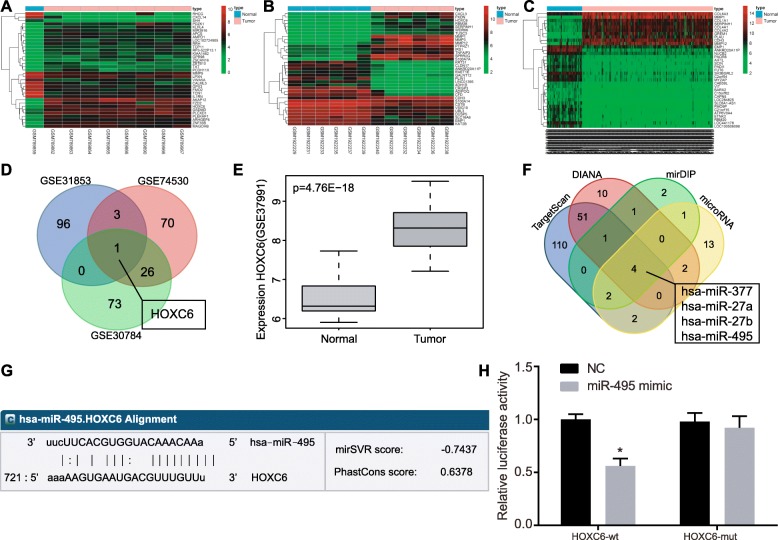
miRNA and mRNA expression profiles in OSCC. **a**–**c** The heat maps of DEGS from the three OSCC-related datasets; the topmost bar represented the sample type, in which blue represented normal control samples, and red represented tumor samples; the color gradation at the upper right represented gene expression, in which the color changed from red to green from the top to the bottom, suggesting gene expression changed from high to low; abscissa represented sample number and the ordinate represented gene names, each rectangle corresponds to a sample expression value. The dendrogram on the left represented cluster analysis based on differences in gene expression. **d** Venn analysis of the top 100 DEGs retrieved from the GSE31853, GSE74530, and GSE30784 datasets; blue indicated the GSE31853 dataset, red indicated the GSE74530 dataset, and green indicated the GSE30784 dataset; blue arrows indicated the intersection of the three datasets. **e** HOXC6 expression analyzed in the GSE37991 dataset; gray boxplot on the left represented HOXC6 expression in 40 normal control samples and dark gray boxplot on the right represented HOXC6 expression in 40 tumor samples with *p* value at the upper left. **f** Predicted regulatory miRNAs of HOXC6 in the four different databases. The predicted results with the highest score were selected for Venn analysis. Blue indicated the TargetScan database predicted results, red indicated the DIANA database predicted results, green indicated the mirDIP database predicted results, yellow indicated the microRNA database predicted results, and blue arrow location represented the intersection of the four database predictions. **g** The predicted binding sites between miR-495 and the 3′-UTR of HOXC6 by an online prediction software. **h** The binding of miR-495 to HOXC6 confirmed by dual-luciferase reporter assay. *p < 0.05 vs. the NC group. Data (mean ± standard deviation) were analyzed by independent sample *t* test. The experiment was repeated 3 times independently

OSCC-related sequencing datasets were retrieved from the GEO database, and three datasets, GSE31853, GSE74530, and GSE30784, were selected. Next, the 3 datasets were subjected to differential analysis, and 284, 374, and 420 OCSS-associated DEGs were found in the GSE31853, GSE74530, and GSE30784 datasets, respectively (Fig. 1a–c). To further screen the OSCC-associated genes, Venn diagrams were constructed by including the top 100 DEGs from the 3 datasets (Fig. 1d). HOXC6 was found to be the only gene that presented in the top 100 DEGs from the 3 datasets, suggesting the potential association of HOXC6 with OSCC. Analysis of HOXC6 expression in these 3 datasets revealed that HOXC6 was highly expressed in OSCC. In order to further confirm the expression of HOXC6 in OSCC, HOXC6 expression was analyzed in the GSE37991 dataset, which also showed the significantly elevated HOXC6 expression in OSCC tissues (Fig. 1e). This result further suggested that HOXC6 was likely to promote OSCC development. Previous studies of OSCC-related signaling pathways revealed that the TGF-β signaling pathway was closely related to OSCC development [18, 19]. However, little is known about whether HOXC6 regulates the TGF-β signaling pathway in OSCC. In order to further understand the mechanism of HOXC6 in OSCC, the potential regulatory miRNAs of HOXC6 were predicted using the TargetScan database, in which the predicted results with “context ++ score percentile” greater than 90 were selected. Based on the predicted results from DIANA database, the scores greater than 0.8 were selected. For prediction using mirDIP, the predicted results with a score greater than 0.5 were selected. Based on the microRNA.org database, a total of 24 regulatory miRNAs were predicted and included for the follow-up analysis. Venn analysis was then conducted for the aforementioned 24 miRNAs and depicted (Fig. 1f) 4 intersected miRNAs, namely hsa-miR-377, hsa-miR-27a, hsa-miR-27b, and hsa-miR-495. Previous studies have highlighted the functional mechanisms of hsa-miR-377, hsa-miR-27a, and hsa-miR-27b in OSCC [20–24], but the studies on the mechanism of hsa-miR-495 in OSCC were limited.

Furthermore, analysis using online bioinformatic software revealed a specific binding region between HOXC6 gene sequence and miR-495 sequence, suggesting that HOXC6 was a target gene of miR-495 (Fig. 1g). Dual-luciferase reporter assay confirmed the binding of miR-495 to HOXC6. As shown in Fig. 1h, compared with the NC group, the miR-495 mimic group showed a significant decrease in the luciferase activity of HOXC6 wild type (Wt) in cells (*p* < 0.05) while there was no significant difference in the luciferase activity of HOXC6-Mut (*p* > 0.05), suggesting that miR-495 could specifically bind to HOXC6. Based on the preliminary analysis from this study, we found that hsa-miR-495 was likely to regulate the expression of HOXC6, thereby affecting the TGF-β signaling pathway and the progression of OSCC.

### Successful isolation of OSCC cells

The relatively purified OSCC cells were obtained by repeated adherence and differential culture for 5 days. Morphological observation results showed that the OSCC cells were tetragonal or polygonal resembling “paving stones” (Additional file 1: Figure S1A). Purified cells were identified by Vimentin and keratin staining using immunohistochemistry. Vimentin was negatively expressed in OSCC cell slides (Additional file 1: Figure S1B) and keratin was positively expressed in OSCC slides (Additional file 1: Figure S1C), suggesting that the purified OSCC cells were successfully obtained. The OSCC cells were then sorted and identified.

### Successful identification of CSCs

Using flow cytometry, CD133 and CD44 positive and negative cells were sorted from OSCC cells (Additional file 2: Figure S2A). Then, the cell spheres enriched in OSCC cells were treated with CSC suspension sphere-forming test in order to identify whether the cell spheres were the CSCs (Additional file 2: Figure S2B). Subsequently, Western blot analysis was performed to detect the expression of stem-related surface molecular markers CD133 and CD44 in OSCC cell spheres, the results of which displayed that the expression of CD133 and CD44 was significantly increased in OSCC cell spheres compared with normal OSCC cells (*p* < 0.05) (Additional file 2: Figure S2C, D). These findings suggested that CSCs were successfully isolated.

### miR-495 inhibits activation of the TGF-β signaling pathway by downregulating HOXC6

**Fig. 2:**
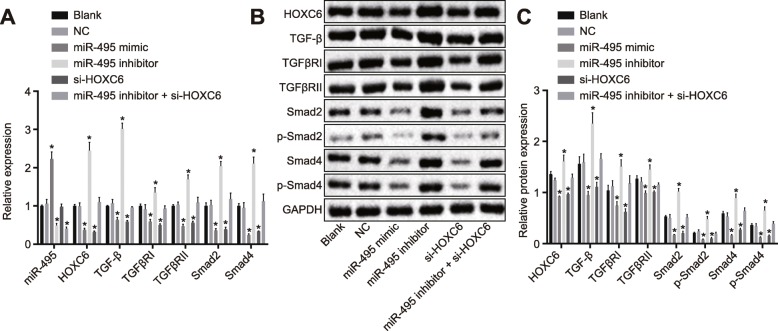
Upregulation of miR-495 or HOXC6 silencing represses the TGF-β signaling pathway. The cells were treated with miR-495 mimic, miR-495 inhibitor, or si-HOXC6. **a** The miR-495 expression and mRNA levels of HOXC6, TGF-β, TGFβRI, TGFβRII, and Smad4 in cells detected by RT-qPCR. **b**, **c**, The protein levels of HOXC6, TGF-β, TGFβRI, TGFβRII, Smad2, and Smad4 in cells examined by Western blot analysis. *p < 0.05 vs. the blank group and the NC group. Data (mean ± standard deviation) were analyzed by independent sample *t* test. The experiment was repeated three times independently

RT-qPCR and Western blot analysis were used to measure the levels of miR-495, HOXC6, TGF-β, TGFβRI, TGFβRII, Smad2, and Smad4 in cells. The results of RT-qPCR are depicted in Fig. 2a. There was no significant difference in miR-495 level and the mRNA level of HOXC6, TGF-β, TGFβRI, TGFβRII, Smad2, and Smad4 between the blank and NC groups (*p* > 0.05). Compared with the blank and NC groups, the mRNA levels of HOXC6, TGF-β, TGFβRI, TGFβRII, Smad2, and Smad4 were significantly lower in the miR-495 mimic group while the expression of miR-495 was higher (*p* < 0.05). Similarly, the levels of HOXC6, TGF-β, TGFβRI, TGFβRII, Smad2, and Smad4 were obviously decreased in the si-HOXC6 group compared to the blank and NC groups (*p* < 0.05), while miR-495 level did not differ significantly (*p* > 0.05). The miR-495 inhibitor group exhibited markedly higher mRNA levels of TGF-β, TGFβRI, TGFβRII, Smad2, and Smad4 yet lower miR-495 level in comparison with the blank and NC groups (*p* < 0.05). In the miR-495 inhibitor + si-HOXC6 group, there was no significant difference in the mRNA levels of HOXC6, TGF-β, TGFβRI, TGFβRII, Smad2, and Smad4 (*p* > 0.05), while miR-495 level was remarkably decreased (*p* < 0.05) compared to the blank and NC groups.

Western blot analysis displayed no significant difference in the protein levels of HOXC6, TGF-β, TGFβRI, TGFβRII, Smad2, and Smad4 as well as the extent of Smad2 and Smad4 phosphorylation between the blank group and the NC group (*p* > 0.05). In comparison with the blank and NC groups, the protein levels of HOXC6, TGF-β, TGFβRI, TGFβRII, Smad2, and Smad4 as well as the extent of Smad2 and Smad4 phosphorylation were diminished in the miR-495 mimic and si-HOXC6 groups (all *p* < 0.05) while an opposite trend was observed in the miR-495 inhibitor group (all *p* < 0.05). The miR-495 inhibitor + si-HOXC6 group showed no changes compared to the blank and NC groups (*p* > 0.05) (Fig. 2b, c). These results demonstrated that the upregulation of miR-495 or downregulation of HOXC6 could inhibit the expression of the TGF-β signaling pathway-related genes in CSCs following OSCC.

### Overexpression of miR-495 or silencing of HOXC6 inhibits EMT in CSCs

**Fig. 3:**
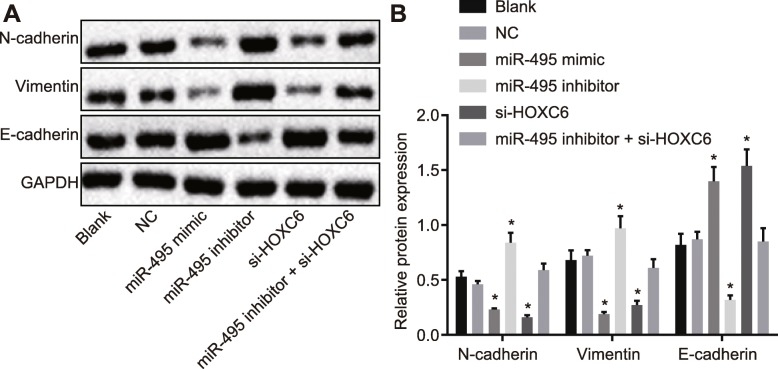
Upregulation of miR-495 or HOXC6 silencing inhibits EMT in CSCs. The cells were treated with miR-495 mimic, miR-495 inhibitor, or si-HOXC6. **a**, **b** The protein expression of N-cadherin, Vimentin, and E-cadherin in CSCs determined by Western blot analysis. *p < 0.05 vs. the blank and NC groups. Data (mean ± standard deviation) were analyzed by independent sample *t* test. The experiment was repeated three times independently

Western blot analysis was carried out to determine the effect of miR-495 and HOXC6 on EMT in CSCs. The results (Fig. 3) showed no significant difference regarding the EMT between the blank group and the NC group (*p* > 0.05). Compared with the blank and NC groups, the miR-495 mimic and si-HOXC6 groups displayed an obvious reduction in the levels of N-cadherin and Vimentin, while a significant increase in E-cadherin level (*p* < 0.05). The miR-495 inhibitor group showed a remarkable increase in the levels of N-cadherin and Vimentin but a reduced E-cadherin level was observed (*p* < 0.05). No evident significance in the EMT was found in the miR-495 inhibitor + si-HOXC6 group relative to the blank and NC groups (*p* > 0.05). The above results led to a conclusion that the overexpression of miR-495 or knockdown of HOXC6 could obstruct EMT in CSCs.

### Overexpression of miR-495 or silencing of HOXC6 inhibits migration, invasion, and proliferation of CSCs in OSCC

**Fig. 4:**
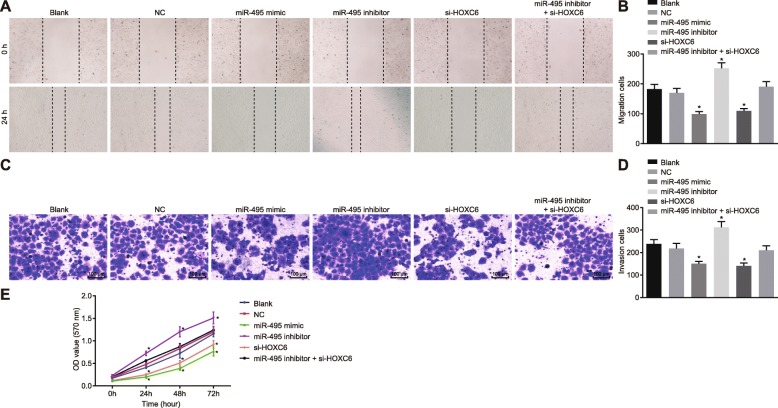
Upregulation of miR-495 or HOXC6 silencing inhibits cell invasion, migration, and proliferation of CSCs in OSCC. The cells were treated with miR-495 mimic, miR-495 inhibitor, or si-HOXC6. **a**, **b** Cell migration ability after different treatments detected by scratch test. **c**, **d** Cell invasion ability after different treatments examined by Transwell assay (× 100). **e** Cell proliferation after different treatments measured by MTT assay. *p < 0.05 vs. the blank group and the NC group. Data (mean ± standard deviation) were analyzed by independent sample *t* test. The experiment was repeated three times independently

Scratch test was performed to detect cell migration in each group, and the results demonstrated that there was no pronounced difference in cell migration between the blank group and the NC group (*p* > 0.05). Compared with the NC and blank groups, the miR-495 mimic and si-HOXC6 groups revealed a significantly reduced cell migration (*p* < 0.05), while the miR-495 inhibitor group exhibited a significantly enhanced cell migration (*p* < 0.05). However, cell migration did not change remarkably in the miR-495 inhibitor + si-HOXC6 group (*p* > 0.05) (Fig. 4a, b).

Transwell assay was performed to test cell invasion in each group, and the results demonstrated that there was no remarkable difference in cell invasion between the blank group and the NC group (*p* > 0.05). Compared with the NC and blank groups, the miR-495 mimic and si-HOXC6 groups displayed a significantly reduced cell invasion (*p* < 0.05), while the miR-495 inhibitor group displayed a significantly elevated cell migration (*p* < 0.05). However, the miR-495 inhibitor + si-HOXC6 group showed no significant changes in cell migration compared with the NC and blank groups (*p* > 0.05) (Fig. 4c, d).

MTT assay was employed to examine the proliferation of CSCs in each group. The results (Fig. 4e) demonstrated that CSC proliferation in each group showed no obvious changes at 0 h (*p* > 0.05). Compared with the cell proliferation at 0 h, the cell proliferation changed remarkably at 24 h, 48 h, and 72 h (*p* < 0.05). Compared with the blank and NC groups, the miR-495 inhibitor group demonstrated significantly faster cell proliferation (*p* < 0.05), while the miR-495 mimic and si-HOXC6 groups showed a significantly slower cell proliferation (*p* < 0.05). The miR-495 inhibitor + si-HOXC6 group exhibited no significant difference in cell proliferation when compared to the blank and NC groups (*p* > 0.05). The above results showed that upregulation of miR-495 or silencing of HOXC6 could inhibit cell proliferation. In sum, overexpression of miR-495 or silencing of HOXC6 exerted an inhibitory effect on CSC cell migration, invasion, and proliferation.

### Overexpression of miR-495 prevents cell cycle entry and promotes CSC apoptosis by suppressing HOXC6

**Fig. 5:**
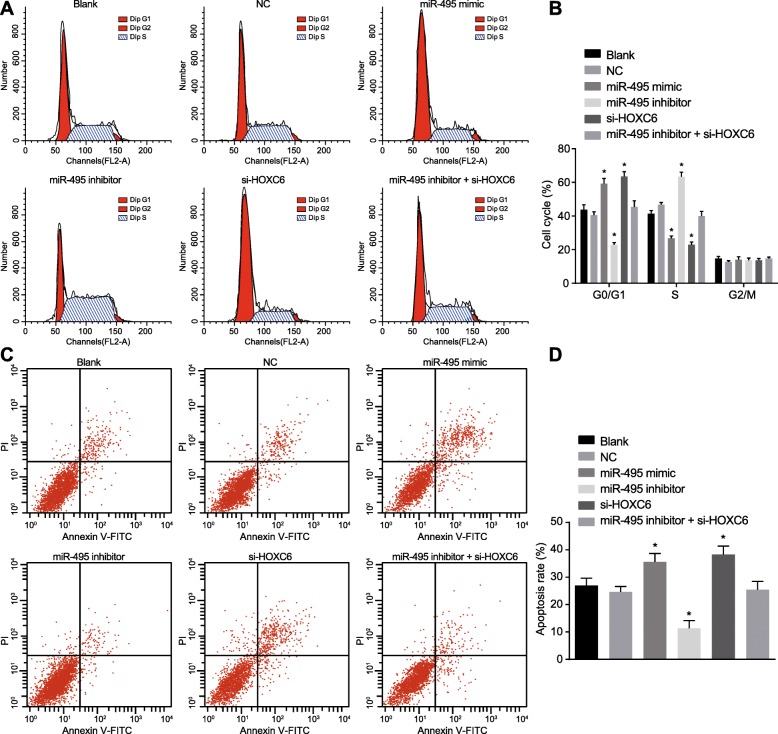
Upregulation of miR-495 or HOXC6 silencing contributes to more cells arrested in the G1 phase and promotes cell apoptosis. The cells were treated with miR-495 mimic, miR-495 inhibitor, or si-HOXC6. **a**, **b** Cell cycle entry after different treatments examined by PI staining. **c**, **d** Cell apoptosis after different treatments detected by Annexin V/PI double staining. Data (mean ± standard deviation) were analyzed by independent sample *t* test. The experiment was repeated three times independently. *p < 0.05 vs. the blank group and the NC group

PI staining was conducted to determine cell cycle distribution in each group. The results (Fig. 5a, b) showed that there was no pronounced difference in the cell cycle between the blank group and the NC group (*p* > 0.05). Compared with the blank and NC groups, the miR-495 mimic and si-HOXC6 groups showed more cells arrested in the G1 phase, while fewer in the S phase (*p* < 0.05), demonstrating that the proliferation of CSCs was significantly inhibited. However, there were fewer cells arrested in the G1 phase and more cells arrested in the S phase in the miR-495 inhibitor group compared to the blank and NC groups (*p* < 0.05). No significant difference was detected in cells arrested in the G1 and S phases among the blank, NC, and miR-300 inhibitor + si-HOXC6 groups (*p* > 0.05).

Annexin V/PI double staining was employed to examine cell apoptosis. The results (Fig. 5c, d) revealed that there was no marked difference in cell apoptosis between the blank group and the NC group (*p* > 0.05). Compared with the blank and NC groups, the cell apoptosis was significantly decreased in the miR-495 inhibitor group (*p* < 0.05), while that was markedly enhanced in the miR-495 mimic and si-HOXC6 groups (*p* < 0.05). However, there was no difference in cell apoptosis among the blank, NC, and miR-495 inhibitor + si-HOXC6 groups (*p* > 0.05). These results suggested that upregulated miR-495 could suppress cell cycle entry and promote cell apoptosis in CSCs by suppressing HOXC6.

### Overexpression of miR-495 or silencing of HOXC6 represses tumor growth in vivo

**Fig. 6:**
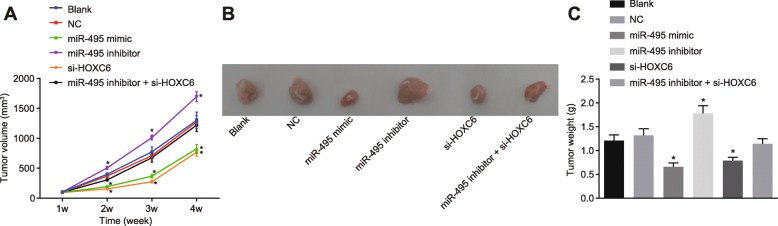
Upregulation of miR-495 or HOXC6 silencing prevents OSCC growth in vivo. The tumor-bearing nude mice were injected with miR-495 agomir, miR-495 antagomir, or sh-HOXC6. **a** The tumor volume of nude mice after injection with lentiviral vectors. **b** The representative images of tumor growth in nude mice after injection with lentiviral vectors at the 28th day. **c** The tumor weight of nude mice after injection with lentiviral vectors. *p < 0.05 vs. the blank group and the NC group. Data (mean ± standard deviation) were analyzed by independent sample *t* test. The experiment was repeated three times independently

Xenograft tumor in nude mice was used to determine the effects of miR-495 and HOXC6 on the tumor growth in vivo. The tumor growth curve illustrated (Fig. 6) that compared with the NC and blank groups, the miR-495 agomir and sh-HOXC6 groups showed delayed tumor growth, while the miR-495 antagomir group demonstrated boosted tumor growth of nude mice after 2 weeks of treatment, with a progressive increase as time went by (*p* < 0.05). After 4 weeks, the mice were euthanized and the tumors were extracted and measured. Compared with the blank and NC groups, the miR-495 agomir and sh-HOXC6 groups displayed significantly decreased tumor volume and weight (*p* < 0.05). The tumor volume and weight were increased in the miR-495 antagomir group in comparison with the blank and NC groups (*p* < 0.05). However, no obvious difference regarding tumor volume and weight was observed in the blank, NC, and miR-495 antagomir + sh-HOXC6 groups (*p* > 0.05). These results showed that miR-495 overexpression could suppress tumor growth in vivo by inhibiting HOXC6.

### Successful transfection of miR-495 interference plasmids

RT-qPCR was used to detect the transfection efficiency of miR-495 overexpression and knockdown. The results (Additional file 3: Figure S3) showed that compared with the NC-mimic group, the expression of miR-495 was increased in the miR-495 mimic group (*p* < 0.05). The expression of miR-495 was lower in the miR-495 inhibitor group than that in the NC-inhibitor group (*p* < 0.05). The above results indicated that miR-495 interference plasmids were successfully constructed.

## Discussion

OSCC represents the 6th most common cancers globally with reported incidence beyond 450,000 cases per annum and with 5-year survival rates of less than 50% [25, 26]. CSCs have drawn increasing attention due to their involvement in the progression of OSCC, and diverse miRNAs have been shown to regulate the cellular functions of CSCs from different types of cancers, such as prostate CSCs, as well as head and neck CSCs [5, 27]. In this study, we examined the potential effects of miR-495 on CSC proliferation, migration, and invasion in OSCC. Our findings confirmed that miR-495 had the capability to inhibit the TGF-β signaling pathway by targeting HOXC6, thereby impairing CSC proliferation, migration, and invasion while inducing cell apoptosis in OSCC.

We initially found that the overexpressed miR-495 markedly inhibited the TGF-β signaling pathway activation, manifested by reduced expression of the pathway-related genes (TGF-β, TGFβRI, TGFβRII, Smad2, and Smad4), by suppressing HOXC6 in CSCs in OSCC. In line with our findings, miR-495 has been found to be downregulated in OSCC tissues and cell lines [28]. In the present study, the in silico analysis indicated that miR-495 could target HOXC6 and then repress its expression in vitro. Upregulation of miR-495 can reduce high glucose-induced inflammatory, cell differentiation, and extracellular matrix accumulation of human cardiac fibroblasts by blocking the nuclear factor-κB (NF-κB) and TGF-β1/Smad signaling pathways [29]. The blockade of HOXC6-dependent TGF-β signaling pathway is responsible for the suppressed EMT and lymph node metastasis in laryngeal cancer [30]. HOXC6 was found to be abundantly expressed in oral cancer FaDu-PTX cells compared to normal cells [31]. Moreover, the activated TGF-β/Smad signaling pathway is capable to promote the proliferation and suppress the apoptosis of OSCC cells [32]. The above findings serve to illustrate an inverse correlation between miR-495 and HOXC6 and TGF-β in OSCC.

Furthermore, forced miR-495 expression or HOXC6 silencing impeded EMT in OSCC cells, evidenced by obviously reduced levels of N-cadherin and Vimentin yet significantly elevated E-cadherin level in the miR-495 mimic and si-HOXC6 groups. EMT is characterized by the loss of epithelial features, and the gain of mesenchymal features has been proposed to offer a connection between cancer metastasis and stem cell properties [1]. The importance of the EMT process in CSC formation has been stressed recently, and miRNAs have also been reported to be involved in controlling CSC functions and regulating cancer progression by affecting the EMT process [33]. Consistently, amplified miR-495 expression has been shown to significantly inhibit EMT-related proteins in OSCC cells in vitro [28]. A previous report showed that HOXB9 may promote OSCC EMT by inducing the activation of the TGF-β1/Smad2/Slug signaling pathway [26]. TGF-β is a key inducer of EMT [34]. The co-stimulation of TGFβ1 and EGF has been revealed to trigger the phenotype transition in OSCC cells, which meets the requirements of EMT accompanied by upregulated Vimentin and downregulated E-cadherin at the protein level [35]. A finding similar to ours was that HOXC6 gene silencing delays EMT (reflected by decreased N-cadherin and Vimentin expression and increased E-cadherin expression) through the inhibition of the activation of TGF-β/Smad signaling pathway in cervical carcinoma cells [14].

Emerging evidence demonstrates that miRNAs play an important role in the regulation of cancer cell growth, invasion, and metastasis by inhibiting the expression of their targets [36]. In the current study, overexpression of miR-495 inhibited the proliferation, migration, and invasion while stimulating the apoptosis of CSCs in OSCC by downregulating its target HOXC6 gene. Similarly, the overexpression of miR-495 inhibits OSCC cell proliferation and invasion through its suppression in Notch1 target gene in vitro [10]. One previous study suggested that 14q32.31 miRNAs, including miR-495, play important roles in the inhibition of cell proliferation, invasion, and migration in metastatic prostate cancer, where miR-495 could decrease cells in proliferative S phase while increase cells in the G1 phase [37]. Another study suggested that the deregulation of HOX genes contributes to cancer development and progression [38]. A previous report revealed that miR-147-mediated HOXC6 silencing can inhibit cell proliferation and migration in hepatocellular carcinoma in vitro [39]. The overexpression of HOXC5 has also found to be associated with oral carcinogenesis and can significantly boost the development of OSCC [40]. Furthermore, siRNA against HOXC6 significantly reduces the growth of xenograft tumors in mice with oral cancer [31] and upregulation of miR-495 can inhibit the growth of OSCC xenografts in vivo [28], which were in agreement with our in vivo experimental results.

## Conclusion

**Fig. 7:**
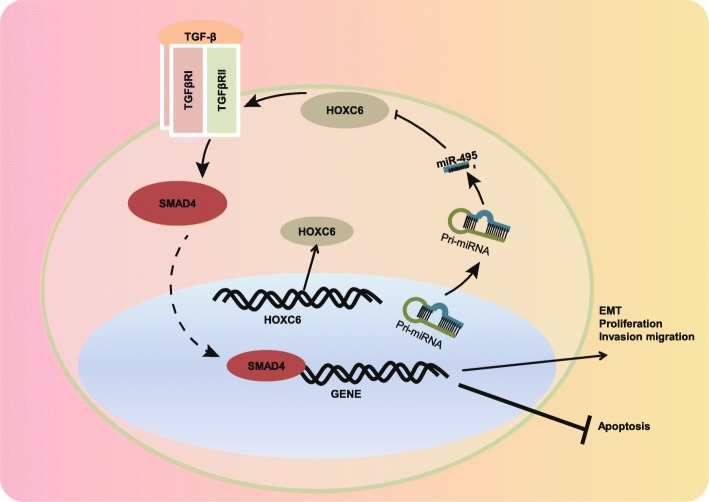
The mechanism graph of the regulatory network of miR-495/HOXC6/TGF-β in the progression of OSCC. miR-495 targets HOXC6 and subsequently represses its expression, leading to the blocked TGF-β signaling pathway, ultimately impeding EMT, proliferation, invasion, and migration of CSCs while promoting cell apoptosis

Our study demonstrates that miR-495 has the potential to retard CSC cell proliferation, invasion, and migration along with EMT while promoting cell apoptosis by perturbing the HOXC6-dependent TGF-β signaling pathway activation (Fig. 7). These findings highlighted the potential of miR-495 as a new therapeutic intervention in the treatment of OSCC. Due to the limited sample size, the exact mechanism of miR-495 is not fully elucidated, and therefore, further large-scale studies are required to illustrate the underlying mechanism.

## Supplementary information

**Additional file 1: Figure S1.** Successful isolation of CSCs. A, The morphological observation of the purified OSCC cells (× 200). B and C, Immunohistochemistry analysis of Vimentin and keratin in OSCC cells (× 200).

**Additional file 2: Figure S2.** Sorting and identification of CSCs. A, The CD133 and CD44 positive cells sorted from OSCC cells detected by flow cytometry. B, The cell spheres enriched in OSCC cells detected by suspension sphere-forming test (× 200). C and D, The protein expression of CD133 and CD44 in OSCC cell spheres examined by western blot analysis. * *p* < 0.05 vs. the OSCC cells. Data (mean ± standard deviation) were analyzed by independent sample *t*-test. The experiment was repeated 3 times independently.

**Additional file 3: Figure S3.** Transfection efficiency of miR-495 overexpression or knockdown. * *p* < 0.05 vs. the NC-mimic group. # *p* < 0.05 vs. the NC-inhibitor group. Data (mean ± standard deviation) were analyzed by independent sample *t*-test. The experiment was repeated 3 times independently.

## Data Availability

The datasets supporting the conclusions of this article are included within the article.

## Abbreviations

3′-UTR: 3′-Untranslated region
ANOVA: Analysis of variance
cDNA: Complementary DNA
CSCs: Cancer stem cells
DEGs: Differentially expressed genes
DMEM: Dulbecco’s modified Eagle’s medium
DMSO: Dimethyl sulfoxide
EGF: Epithelial growth factor
EMT: Epithelial-mesenchymal transition
FBS: Fetal bovine serum
FITC: Fluorescein isothiocyanate
GAPDH: Glyceraldehyde-3-phosphate dehydrogenase
GEO: Gene Expression Omnibus
HOXC6: Homeobox C6
IMDM: Iscove’s modified Dulbecco’s medium
LSD: Least significant difference
MEM: Minimum essential medium
NC: Negative control
NF-κB: Nuclear factor-κB
OD: Optical density
OSCC: Oral squamous cell carcinoma
PBS: Phosphate buffer saline
PMSF: Phenylmethanesulfonyl fluoride
PVDF: Polyvinylidene fluoride
RIPA: Radio-immunoprecipitation assay
RT-qPCR: Reverse transcription quantitative polymerase chain reaction
SDA-PAGE: Sodium dodecyl sulfate polyacrylamide gel electrophoresis
sh: Short hairpin RNA
si: Small interfering RNA
SP: Streptavidin peroxidase
SPF: Specific pathogen-free
TBST: Tris-buffered saline with Tween-20
TGF-β: Transforming growth factor-beta
wt: Wild type
